# Explainable Machine Learning Predictions for the Benefit From Chemotherapy in Advanced Non‐Small Cell Lung Cancer Without Available Targeted Mutations

**DOI:** 10.1111/crj.70044

**Published:** 2024-12-18

**Authors:** Zhao Shuang, Xiong Xingyu, Cheng Yue, Yu Mingjing

**Affiliations:** ^1^ Department of Respiratory and Critical Care Medicine, State Key Laboratory of Respiratory Health and Multimorbidity West China Hospital, Sichuan University Chengdu Sichuan China

**Keywords:** chemotherapy, machine learning, NSCLC, outcome, survival

## Abstract

**Background:**

Non‐small cell lung cancer (NSCLC) is a global health challenge. Chemotherapy remains the standard therapy for advanced NSCLC without mutations, but drug resistance often reduces effectiveness. Developing more effective methods to predict and monitor chemotherapy benefits early is crucial.

**Methods:**

We carried out a retrospective cohort study of NSCLC patients without targeted mutations who received chemotherapy at West China Hospital from 2009 to 2013. We identified variables associated with chemotherapy outcomes and built four predictive models by machine learning. Shapley additive explanations (SHAP) interpreted the best model's predictions. The Kaplan–Meier method assessed key variables' impact on 5‐year overall survival.

**Results:**

The study enrolled 461 NSCLC patients. Eight variables were selected for the model: differentiation, surgery history, neutrophil‐to‐lymphocyte ratio (NLR), platelet‐to‐lymphocyte ratio (PLR), total bilirubin (TBIL), total protein (TP), alanine aminotransferase (ALT), and lactate dehydrogenase (LDH). The extreme gradient boosting (Xgboost) model exhibited superior discriminatory ability in predicting complete response (CR) probabilities to chemotherapy, with an AUC of 0.78. SHAP plots showed surgery history and high differentiation were related to CR benefits from chemotherapy. Absence of surgery, higher NLR, higher PLR, and higher LDH were all independent prognostic factors for poor survivals in NSCLC patients without mutations receiving chemotherapy.

**Conclusions:**

By machine learning, we developed a predictive model to assess chemotherapy benefits in NSCLC patients without targeted mutations, utilizing eight readily available and non‐invasive clinical indicators. Demonstrating satisfactory predictive performance and clinical practicability, this model may help clinicians identify patients' tendency to benefit from chemotherapy, potentially improving their prognosis.

## Graphical abstract

In this retrospective cohort study, four machine learning models using readily available clinical features were developed to predict chemotherapy outcomes for NSCLC patients without targeted mutations. The Xgboost model, with its strong predictive performance and clinical utility, may assist clinicians in early identification of patients likely to benefit from chemotherapy, potentially improving their prognosis.

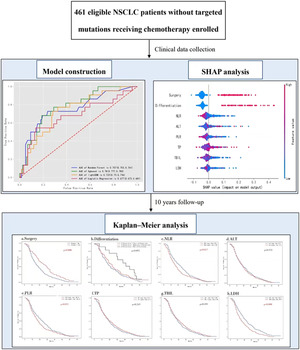

## 1 Introduction

Non‐small cell lung cancer (NSCLC) is a major histologic subtype in lung cancer, comprising approximately 85% of all the cases [1]. The 5‐year overall survival (OS) rate for patients with advanced NSCLC is generally dismal, as over half of these patients present with distant metastasis at the time of diagnosis [2, 3]. Advanced NSCLC is usually classified as an unresectable disease, either locally advanced (Stage III) or metastatic to distant sites (Stage IV) [4, 5]. The prognosis for advanced NSCLC is poor because there is no curative therapy available for these patients; the main treatment goal is to prolong patients' survival.

Although immunotherapy and targeted therapy have achieved remarkable progress in the treatment of NSCLC, many patients have no available targeted mutations. Thus, chemotherapy still remains a dominant standard therapy for the first‐ and second‐line treatment of advanced NSCLC without mutations. Additionally, in many instances, cancer cells evolve to resist drugs, rendering chemotherapy ineffective [6]. Consequently, it is necessary to explore more effective, practical, and non‐invasive methods for predicting and monitoring chemotherapy benefits at an early stage in clinical practice.

The mechanisms underlying drug resistance to chemotherapy are multifaceted and not fully understood [7]. To manage the whole‐course chemotherapy appropriately, a precise prediction model is necessary to identify patients who will benefit from chemotherapy and to optimize the therapeutic strategies. Several prediction models have been previously published and utilized during chemotherapy. For example, Brooks' study employed the C‐statistic model to evaluate the risk of hospitalization among cancer patients following the initiation of chemotherapy [8]. The study found that the model's C‐statistic was 0.69 (95% CI 0.62–0.75) in the validation cohort. Depending on the prespecified threshold, 39% of patients were identified as high‐risk. The 1‐month hospitalization risk was 8.7% (95% CI 6.1%–12.0%) in the low‐risk group and 24.2% (95% CI 19.9%–32.0%) in the high‐risk group. Moreover, mathematical modeling and subsequent analysis have become increasingly valuable for determining the optimal conditions for specific treatment strategies and numerically refining treatment regimens [9]. Despite these advancements, the application of machine learning in the field of lung cancer remains limited.

Machine learning algorithms are increasingly recognized in the literature for their applications in interpreting medical images, diagnosing conditions, predicting outcomes, and planning treatments [10, 11, 12]. Unlike traditional methods, machine learning does not rely on predefined assumptions about input variables and their correlations with the outputs. This data‐driven approach, free from rules‐based programming, makes machine learning a powerful and practical method.

Thus, our study utilized explainable machine learning techniques to create a model aimed at accurately predicting the benefits of chemotherapy for NSCLC patients without available targeted mutations. Baseline variables and physiological data were employed to enhance and fine‐tune the prediction model.

## 2 Methods

### 2.1 Study Participants and Follow Up

We retrospectively included 461 NSCLC patients who were hospitalized at West China Hospital from January 2009 to December 2013. Their medical records were reviewed by two independent authors. The inclusion criteria were as follows: (1) diagnosed NSCLC confirmed by biopsy; (2) genetic testing revealed no available targeted mutations (such as EGFR, ALK, ROS1, RET, MET, BRAF, and HER2); (3) availability of data on blood chemistries, complete blood count, and other inflammatory markers within 1 week prior to the first cycle of chemotherapy. (4) Clinical staging was Stage III or IV based on the TNM classification of lung cancer.

Follow‐ups were conducted every 3 months via telephone, focusing on the tumor progression, recurrence, metastasis, and survival duration. All information was recorded anonymously to ensure patient confidentiality. Short‐term outcomes were evaluated as complete response (CR), partial response (PR), stable disease (SD), and progressive disease (PD) according to the Response Evaluation Criteria in Solid Tumors (RECIST 1.1). The survival status was recorded as the last follow‐up in November 2023. The OS duration was considered to be from the date of confirmed NSCLC diagnosis to the date of death, or to the latest follow‐up of patients who were still alive when the data was censored. This research was approved by the Ethical Committee of West China Hospital, Sichuan University. All enrolled participants signed informed consent before their involvement in the research.

### 2.2 Data Collection

The data were retrospectively obtained from electronic medical records, comprising gender, age, smoking history, pathological subtype, clinical stage, differentiation, therapy, and laboratory results. Clinical staging was according to the 8th edition of the American Joint Committee on Cancer Staging System (AJCC) [4].

### 2.3 Statistical Analysis and Machine Learning Models

Statistical analyses were conducted utilizing Python (Version 3.11.5). Categorical variables were analyzed with the chi‐square test and presented as percentages and frequencies. Continuous variables were reported as mean ± standard deviation for normally distributed data and as median with interquartile range for non‐normally distributed data. Comparisons between two independent sample groups were conducted using two‐sample *t*‐tests when the variables were normally distributed and the Kruskal–Wallis test when the variables were not normally distributed. In all analyses, a *p*‐value below 0.05 was considered to indicate statistical significance.

Four well‐recognized algorithms were employed, including random forest (RF) [13], extreme gradient boosting (Xgboost) [14], light gradient boosting machine (LightGBM) [15], and logistic regression (LR) [16]. The performance of each model was assessed utilizing key statistical metrics, including the receiver operating characteristic (ROC) curve and the area under the curve (AUC), to evaluate the model's discriminative capability. In addition, the models were evaluated based on sensitivity and specificity to measure their accuracy in predicting positive and negative cases, respectively. The positive predictive value (PPV) and negative predictive value (NPV) were assessed as well, in order to further understand the reliability of the model prediction. These indicators collectively offer a comprehensive overview of each model's prediction performance.

Then, we employed various visualization tools of Shapley additive explanations (SHAP) to interpret the prediction for our model more clearly [17]. These tools included the SHAP summary plots, dependence plots, and force plots. The SHAP summary plot visualizes the importance and impact for each variable in all samples, with each dot representing a SHAP value for a feature and an instance. The color coding (blue for low values and red for high values) aids in understanding how feature values affect the model output [18]. The SHAP dependence plot provides insights into how individual feature value affects the prediction. It depicts the SHAP value of a single feature against its actual value for all instances, highlighting the trend in how this feature influences the model's decisions. For individual prediction explanations, the force plots illustrate how each variable alters the output of the model from a baseline value to the actual prediction. Variables that increase the prediction are shown in red, whereas those that decrease it are displayed in blue. These visualization tools were crucial in identifying the determinants of chemotherapy response in NSCLC patients without mutations, revealing how factors influence chemotherapy efficacy.

Survival analysis was implemented utilizing the Kaplan–Meier method for estimating the survival functions from lifetime data. Differences in survival rates between groups, based on clinical and biochemical factors, were evaluated utilizing log‐rank analysis.

## 3 Results

### 3.1 Patient Characteristics

A total of 461 patients with NSCLC were included in our analysis. In order to assess benefits from chemotherapy, we divided all enrolled patients into two groups depending on their chemotherapy response: the CR group and the PR + SD + PD group. Table 1 presents the initial clinical characteristics for the patients. The median age for the participants was 59.2 years, with 148 females and 313 males. Among all the patients, 252 had a history of smoking. The CR and PR + SD + PD groups did not show any significant differences in pathological type and radiotherapy (all *p* > 0.05). However, a higher proportion of Stage IV patients were observed in the PR + SD + PD group (*n* = 258, 66.5%) than the CR group (*n* = 36, 49.3%) (*p* = 0.008). Additionally, a higher percentage of patients in the CR group underwent surgery (*n* = 42, 57.5%) compared with the PR + SD + PD group (*n* = 59, 15.2%) (*p* < 0.001). The median survival period for the patients was 16 (9, 29) months, while only 15 (8, 26) months of PR + SD + PD group and 27 (16, 43) months of CR group (*p* < 0.001).

**TABLE 1:** Baseline and clinical characteristics of patients.

	Overall (*n* = 461) *n* (%) or median (IQR or SD)	PR + SD + PD (*n* = 388) *n* (%) or median (IQR or SD)	CR (*n* = 73) *n* (%) or median (IQR or SD)	*p*‐value
Age (years)	59.189 (11.754)	59.577 (11.742)	57.123 (11.682)	0.103
Gender				
Male	313 (67.896%)	266 (68.557%)	47 (64.384%)	0.573
Female	148 (32.104%)	122 (31.443%)	26 (35.616%)	
Smoking history				
Yes	252 (54.664%)	218 (56.186%)	34 (46.575%)	0.166
No	209 (45.336%)	170 (43.814%)	39 (53.425%)	
Pathological type				0.221
ADC	237 (51.41%)	204 (52.58%)	33 (45.21%)	
SCC	132 (28.63%)	104 (26.8%)	28 (38.36%)	
Others	92 (19.96%)	80 (20.62%)	12 (16.44%)	
Clinical stages				0.008*
III	167 (36.226%)	130 (33.505%)	37 (50.685%)	
IV	294 (63.774%)	258 (66.495%)	36 (49.315%)	
Differentiation				<0.001*
Well	12 (2.6%)	3 (0.77%)	9 (12.33%)	
Moderate	61 (13.23%)	34 (8.76%)	27 (36.99%)	
Poor	128 (27.77%)	102 (26.29%)	26 (35.62%)	
Unknown	260 (56.4%)	249 (64.18%)	11 (15.07%)	
Surgery				<0.001*
Yes	101 (21.909%)	59 (15.206%)	42 (57.534%)	
No	360 (78.091%)	329 (84.794%)	31 (42.466%)	
Radiotherapy				0.556
Yes	34 (7.38%)	27 (6.96%)	7 (9.59%)	
No	402 (87.20%)	341 (87.89%)	61 (83.56%)	
Unknown	25 (5.42%)	20 (5.15%)	5 (6.85%)	
Laboratory tests				
Hemoglobin (g/L)	129.542 (18.075)	129.531 (18.313)	129.603 (16.869)	0.974
Platelet (× 109/L)	213.701 (84.957)	215.822 (86.283)	202.425 (77.098)	0.184
Leukocyte (× 109/L)	7.331 (3.275)	7.418 (3.395)	6.873 (2.516)	0.113
Neutrophil (× 109/L)	5.103 (2.875)	5.202 (2.970)	4.576 (2.249)	0.041*
Lymphocyte (× 109/L)	1.516 (0.568)	1.497 (0.562)	1.615 (0.589)	0.115
NLR	3.881 (2.988)	3.989 (3.045)	3.307 (2.613)	0.049*
PLR	161.027 (94.062)	164.059 (94.831)	144.910 (88.752)	0.097
TP	66.814 (6.095)	66.665 (5.999)	67.585 (6.561)	0.269
Albumin (g/L)	38.773 (4.796)	38.665 (4.800)	39.338 (4.768)	0.272
Globulin (g/L)	28.040 (4.888)	28.001 (4.835)	28.247 (5.187)	0.708
TBIL	10.836 (4.991)	10.989 (5.110)	10.037 (4.265)	0.094
ALT	26.843 (23.914)	26.702 (23.734)	27.575 (24.983)	0.783
AST	24.803 (13.717)	24.982 (14.088)	23.877 (11.644)	0.475
Urea	5.360 (2.048)	5.381 (2.087)	5.250 (1.842)	0.588
CRE	74.112 (28.318)	74.204 (30.101)	73.632 (16.313)	0.816
LDH (U/L)	208.134 (104.182)	212.362 (109.431)	186.056 (67.052)	0.008*
CEA (ng/mL)	5.170 [2.310, 20.005]	5.455 [2.415, 21.945]	4.190 [1.645, 8.065]	0.022*
CYRF21‐1 (ng/mL)	4.850 [2.958, 9.163]	5.125 [3.118, 9.488]	3.695 [2.480, 6.562]	0.057
Overall survival (month)	16.000 [9.00, 29.000]	15.000 [8.000, 26.000]	27.000 [16.000, 43.000]	< 0.001*

Abbreviations: ADC, adenocarcinoma; ALT, alanine aminotransferase; AST, aspartate aminotransferase; CEA, carcino‐embryonic antigen; CR, complete response; CRE, creatinine; CYRF21‐1, cytokeratin fragment antigen 21‐1; LDH, lactate dehydrogenase; NLR, neutrophil‐to‐lymphocyte ratio; PD, progressive disease; PLR, platelet‐to‐lymphocyte ratio; PR, partial response; SCC, squamous cell carcinoma; SD, stable disease; TBIL, total bilirubin; TP, total protein.

* *p*‐value < 0.05.

### 3.2 Model Construction and Interpretation

**FIGURE 1:**
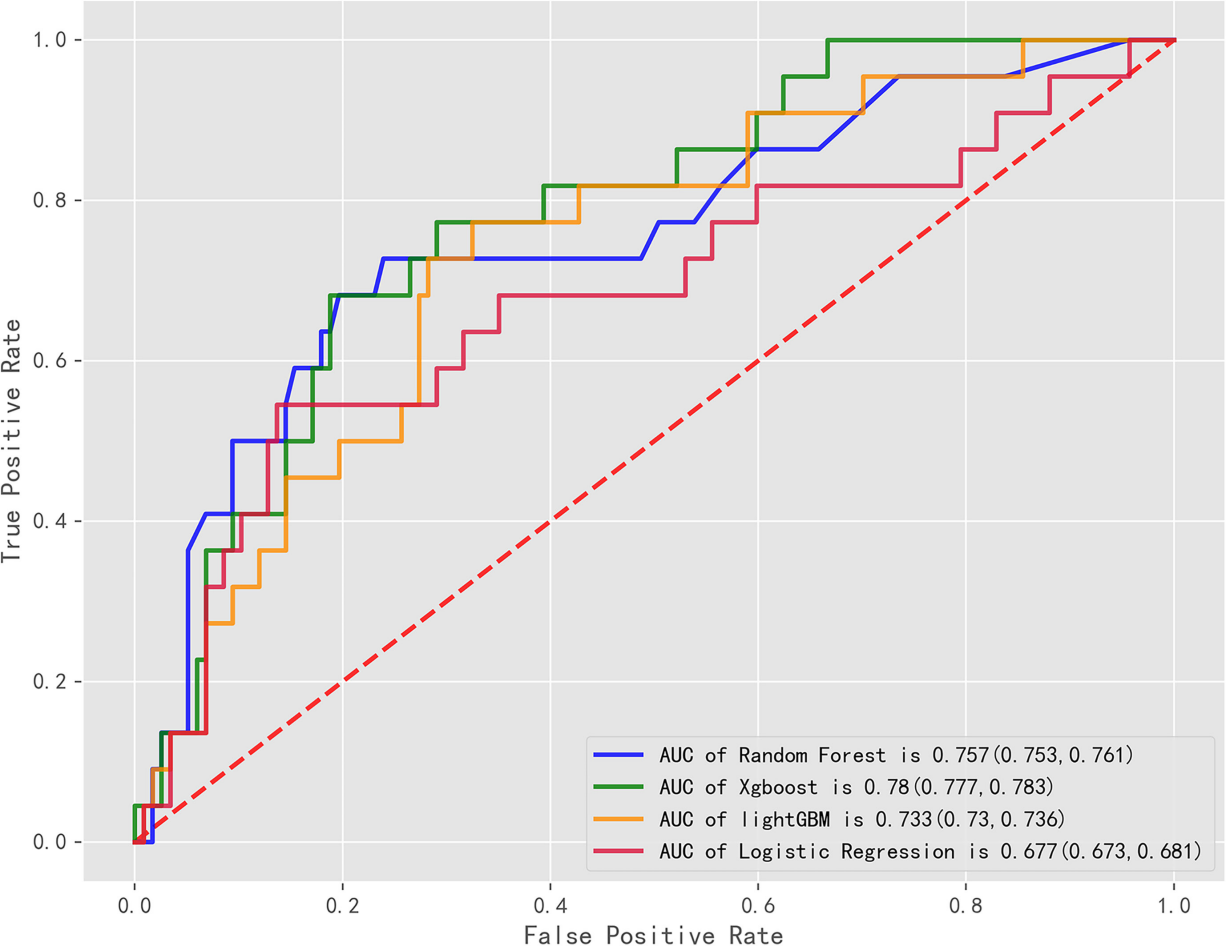
Comparison of area under the curves (AUCs) among machine learning models.

Based on the LASSO algorithm (Figure S1) and clinical utility, we developed final machine learning methods incorporating eight variables, including differentiation, surgery, neutrophil‐to‐lymphocyte ratio (NLR), platelet‐to‐lymphocyte ratio (PLR), total bilirubin (TBIL), total protein (TP), alanine aminotransferase (ALT), and lactate dehydrogenase (LDH). As illustrated in Figure 1 and detailed in Table S1, all models demonstrated acceptable performance in predicting the benefits of chemotherapy for NSCLC without available targeted mutations. Among the single models, Xgboost showed the highest efficacy in forecasting the outcome, with an AUC of 0.78 (95% CI 0.777–0.783).

### 3.3 Individual Variable Impact

**FIGURE 2:**
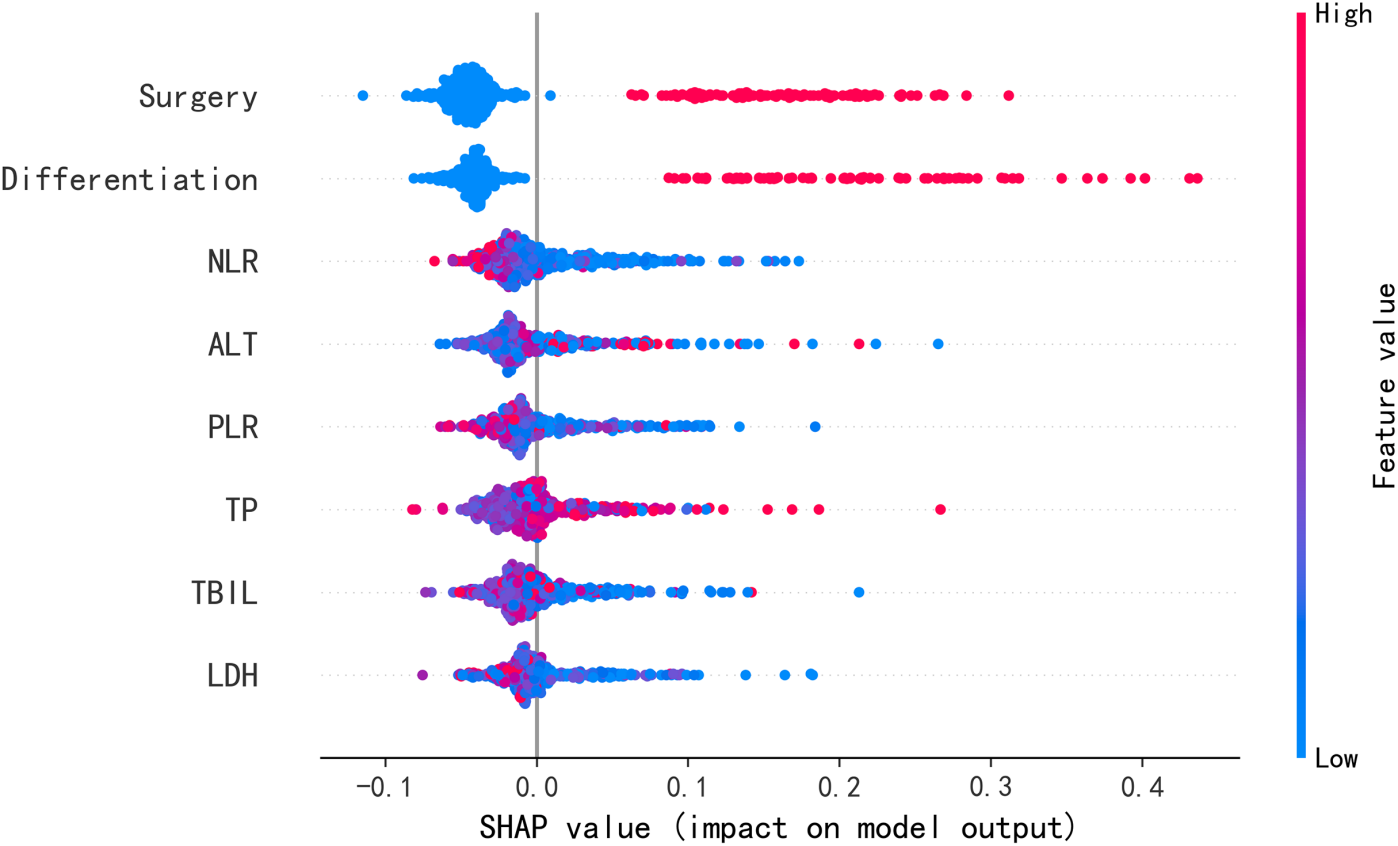
SHAP summary plot of the eight features of the Xgboost model. The higher the SHAP value of a feature, the higher the probability of complete response for chemotherapy.

**FIGURE 3:**
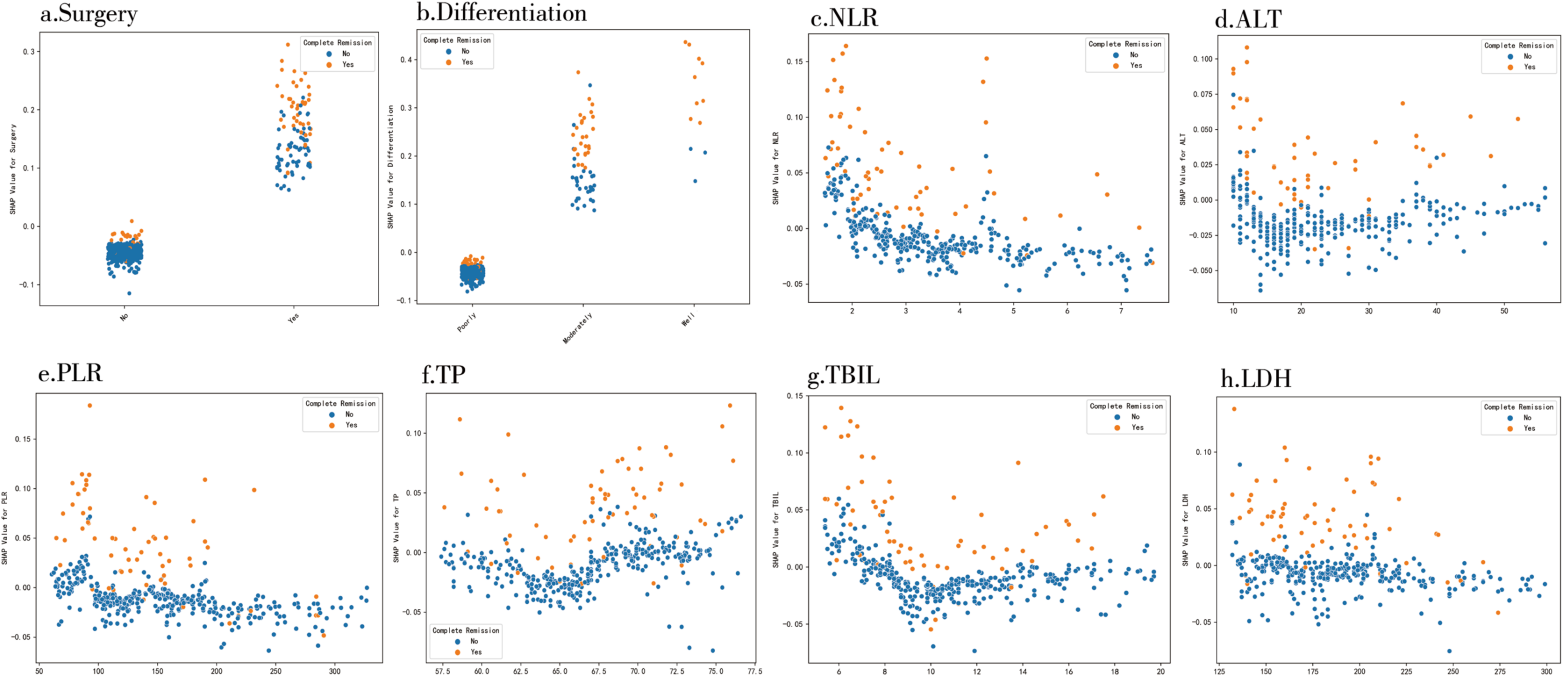
SHAP dependence plot of the Xgboost model. a. surgery; b. differentiation; c. NLR; d. ALT; e. PLR; f. TP; g. TBIL; h. LDH.

A SHAP summary plot was employed for the Xgboost model to further identify the impact of each variable for the model (Figure 2). In this plot, each dot represents a feature attribution value for an individual patient's model, with one dot corresponding to each feature per patient. The dots are colored according to the feature values for each patient, with blue for lower values and red for higher values. The dots accumulate vertically to show density. Among all variables, surgery ranked highest, followed by differentiation, NLR, ALT, PLR, TP, TBIL, and LDH. Then, we conduct a SHAP dependence plot (Figure 3) to intuitively understand how individual features influence the Xgboost prediction model's output. SHAP values greater than zero for specific variables indicate a higher risk for CR. A history of surgery and high degree of differentiation were related to CR benefits of chemotherapy for NSCLC patients without mutations, whereas higher NLR, higher ALT, higher PLR, lower TP, higher TBIL, and higher LDH were associated with worse benefits from chemotherapy.

### 3.4 SHAP Explanation

**FIGURE 4:**
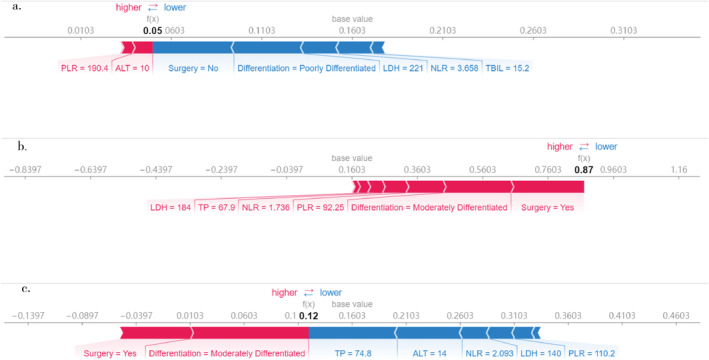
Feature importance of three example individuals explained by SHAP. The model output value is the algorithm score for these three individual example patients. The red colors indicate that a feature pushed the output to a higher score, and blue colored features reduced the output score. a. false claim Patient #1, b. true claim Patient #2, c. false claim Patient #3.

Then, to further illustrate the SHAP explanation, we chose three patients randomly, as listed in Figure 4. The figure displays the various variables contributing to deviations from the base probability value towards the predicted truth probability. Features in red push the prediction towards being true, while those in blue reduce the probability of truth. The first example, shown in Figure 4a, depicts a false claim with a probability of 0.05 for predicting CR responses for chemotherapy. The factors influencing this prediction include surgery, differentiation, LDH, NLR, and TBIL. Clinically, the patient's response to chemotherapy was PD, and OS was 6 months. The second example, depicted in Figure 4b, illustrates a true claim with a probability of 0.87 for predicting CR responses. Contributing factors include surgery, differentiation, PLR, NLR, TP, and LDH. Clinically, this patient achieved a CR to chemotherapy, with an OS of 59 months. The third example (see Figure 4c) presents a false claim with a probability of 0.12 for predicting CR responses. Influential factors here are TP, ALT, NLR, LDH, and PLR. Clinically, the patient's chemotherapy response was SD, and the OS was 7 months. These examples elucidate how specific features drive the model's predictions, enhancing its transparency and trustworthiness.

### 3.5 Survival Analysis

**FIGURE 5:**
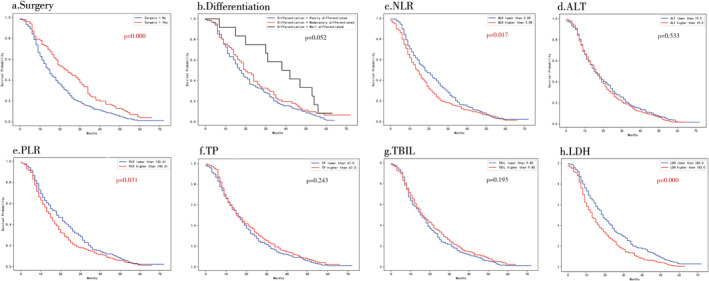
Kaplan–Meier curves for patients' overall survival of the eight features of the Xgboost model. a. surgery; b. differentiation; c. NLR; d. ALT; e. PLR; f. TP; g. TBIL; h. LDH.

Finally, we used the Kaplan–Meier method to further evaluate the effects of these eight variables on patients' 5‐year OSs. As shown in Figure 5, the absence of surgery, higher NLR, higher PLR, and higher LDH were all independent prognostic factors for poor survival in NSCLC patients without available targeted mutations receiving chemotherapy (all *p* < 0.05). Nevertheless, differentiation, ALT, TP, and TBIL all had no significant correlations with patients' 5‐year OSs (all *p* > 0.05).

## 4 Discussion

In our retrospective cohort study, we created four machine learning models to predict chemotherapy outcomes for NSCLC patients without available targeted mutations, utilizing non‐invasive and readily accessible clinical features. Among these models, the Xgboost model demonstrated the highest discrimination power in predicting CR probabilities for chemotherapy. This model can be a promising tool for identifying patients who tend to benefit from chemotherapy either before or early in the treatment process, potentially improving their prognosis.

So far, a few pieces of literature have applied machine learning models to predict treatment outcomes in lung cancer, encompassing immunotherapy [19, 20] and targeted therapy [21, 22]. However, a significant number of patients lack targetable mutations. For these individuals, chemotherapy still remains the predominant treatment. Thus, we specifically address this patient group in our study. Research on employing machine learning to predict treatment outcomes for lung cancer patients without targeted mutations is limited. For example, a machine learning re‐analysis of the NADIM cohort (NCT03081689) using an Xgboost algorithm yielded an AUC of 0.69 in predicting the pathological CR of neoadjuvant chemo‐immunotherapy in resectable Stage IIIA NSCLC [23]. Another study using a radiomic model based on initial CT features before chemotherapy reported an AUC of 0.941 (95% CI 0.898–0.982) and accuracy of 85.7% [24]. Additionally, some studies have incorporated a broader range of indicators to establish their models. Sun et al. used metabolomics combined with machine learning to find promising biomarkers for diagnosing NSCLC and evaluating pemetrexed treatment efficacy, achieving an AUC of 0.981 for the diagnosis of NSCLC and 0.954 for the efficacy of pemetrexed [25]. In our study, we selected non‐invasive and easily accessible clinical characteristics and laboratory results, making our model more applicable in clinical practice. Among these models, the Xgboost model demonstrated the most reliable discrimination power, with an AUC of 0.78, specificity of 73.5%, and sensitivity of 69.8%. Its high NPV of 92.8% suggests it could be employed in identifying patients unlikely to benefit from chemotherapy, allowing clinicians to switch to alternative treatments more promptly.

We selected eight predictors using the LASSO algorithm and clinical utility: surgery history, differentiation, NLR, ALT, PLR, TP, TBIL, and LDH. ROC analysis indicated that all four models had acceptable accuracy in predicting chemotherapy outcomes, with AUCs ranging from 0.677 to 0.780, and outperformed traditional LR models. The Xgboost model reached the best AUC of 0.78, highlighting its potential as a clinical decision‐support tool.

Previous research corroborates the predictive factors we selected. Among them, NLR is the most frequently reported. A small‐scale study of 52 patients indicated that elevated NLR was relevant to lower response rates and worse OS in NSCLC patients receiving nivolumab [26]. For neoadjuvant chemotherapy or combination with immunotherapy in NSCLC, the study also revealed that elevated NLR levels were related to poor pathological response and reduced PFS [27]. Conversely, a large‐scale study (*n* = 934) demonstrated that pretreatment NLR and PLR were related to chemotherapy outcomes and prognosis in lung cancer patients [28]. However, some studies reported inconsistent results. In patients with extensive‐stage SCLC, there was no significant difference in median PFS between those with low pretreatment NLR (NLR ≤ 3.17) and those with high NLR (6.2 vs. 5.8 months; *p* = 0.675) [29]. A South Korea study suggested that in the non‐survival group, baseline levels of NLR, LDH, and CRP were higher, but only LDH and CRP levels were significant indicators for OS in NSCLC patients receiving immunotherapy [30]. Our findings revealed that higher NLR, PLR, and LDH were all independent prognostic indicators for poor survival in NSCLC patients without available targeted mutations receiving chemotherapy. The varied results among studies may stem from differences in sample size, type of lung cancer, and treatment protocols, underscoring the need for more precise patient selection and tailored multimodal treatment strategies.

We applied a SHAP analysis to the Xgboost model to assess the impact of each variable. SHAP values measure the impact of each variable on the risk of CR in these patients. We found that a history of surgery ranked highest among all variables. Specifically, a history of surgery was related to CR benefits from chemotherapy for NSCLC patients without mutations. Notably, Kaplan–Meier analysis suggested that having no surgery history was an independent prognostic indicator for poor OS in these patients. Locally advanced NSCLC is a heterogeneous group of tumors. In most previous studies, it is believed that upfront surgery is a viable option for managing Stage IIIA/B NSCLC [31]. A multimodal treatment approach, which includes surgery for carefully selected patients, has been shown to improve survival rates compared with non‐surgical definitive therapy [32, 33]. For Stage IV NSCLC, there commonly is no opportunity for surgery. However, some studies have suggested that surgery can still offer certain benefits for lung cancer patients even with Stage IV. In a cohort study of advanced NSCLC patients from the National Cancer Database, researchers employed Cox proportional hazards models to evaluate the effectiveness of surgical selection score (SSS) for OS and the impact of surgery on survival across different stages for patients with high SSS levels. They analyzed data from 300 572 patients, of whom 18 701 (6%) underwent surgery. The results revealed a statistically significant improvement in OS among those who underwent surgery (*p* < 0.001). For patients in the upper quartile of SSS without surgery, the risk of death was more than double compared with those who did, even after adjusting for SSS. This increased risk was consistent across different stages: Stage IIIA (HR 2.1; 95% CI 2.0–2.2), Stage IIIB (HR 2.3; 95% CI 2.2–2.5), and Stage IV (HR 2.3; 95% CI 2.2–2.4). These findings underscore the importance of a thorough evaluation of the potential benefits of surgical resection in advanced‐stage patients [34]. Additionally, salvage surgery after downstaging advanced NSCLC through neoadjuvant therapy is a potential treatment strategy for some Stage IIIB–IV NSCLC patients, which could significantly prolong PFS compared with having no salvage surgery [35]. A cohort study enrolled 9 Stage III and 20 Stage IV patients who underwent rescue surgery due to severe, non‐manageable tumor‐related complications (such as super‐infected tumor necrosis, post‐obstructive pneumonia, or active bleeding). The 1‐year survival rate was 45.2%, and mean OS time was 13.3 months. However, the mortality rates at 30 and 90 days were 13.3% and 30%, respectively, indicating that rescue surgery for lung cancer patients with advanced stage is related to significant morbidity and mortality [36]. Thus, for advanced‐stage patients, multidisciplinary team collaboration is increasingly vital to improve patient survival outcomes.

In the final section of our results, we randomly selected three observations to illustrate how SHAP values elucidate individual predictions. Different features influence deviations from the baseline probability towards the predicted probability of the true outcome. The results demonstrate that the model variables are accessible, and the prediction method is clear and comprehensible. Furthermore, these findings align well with the real‐world clinical efficacy and prognosis of the patients. This model offers promising prospects for clinical applications. In future clinical settings, for Stage III or IV NSCLC patients lacking targeted mutations, before they commence chemotherapy, a prediction result about the efficacy of chemotherapy can be obtained through this model. For those patients who might have suboptimal responses to chemotherapy, physicians can consider more combined treatment modalities like combined immunotherapy, anti‐angiogenesis therapy, or concurrent radiotherapy when making treatment decisions. Naturally, this supposition still demands more clinical investigations for verification.

Although our model demonstrates strong predictive performance and substantial practicality, this study has several limitations. Firstly, the study had a relatively small sample size at a single center. Secondly, this study is a retrospective study, which cannot completely exclude confounding factors, leading to lower reliability of the conclusions. Thus, further multi‐center prospective study is needed to verify its reliability. Thirdly, despite the strong prognostic capabilities of our model in internal validation, no external validation was performed to confirm its effectiveness in different settings. Fourthly, while the algorithm effectively learned from the input features, it may have overlooked or failed to identify some hidden relationships due to neglected or unknown factors.

In conclusion, we constructed a prediction model for chemotherapy benefits for NSCLC patients without targeted mutations using eight readily available and non‐invasive clinical indicators. Demonstrating satisfactory predictive performance and clinical practicability, this model can help clinicians identify patients' tendency to benefit from chemotherapy either before or early in the treatment process, potentially improving their prognosis. However, multi‐center prospective research is needed to further confirm the model's efficacy and safety before routine clinical practice.

## Author Contributions

**Zhao Shuang:** design, draft writing, review and editing, analysis, methodology, project administration. **Xiong Xingyu:** design, review and editing, method writing, analysis, methodology, validation. **Cheng Yue:** data collection and curation, investigation. **Yu Mingjing:** revise and polish the article to enhance its quality.

## Ethics Statement

This research was conducted in compliance with ethical standards and guidelines for research involving human participants. This research was approved by the Ethical Committee of West China Hospital, Sichuan University (Ethics Approval Number: 2018 Review No. 59). All enrolled participants wrote an informed consent before their involvement in the research. Measures were taken to ensure confidentiality and anonymity of participants' data.

## Conflicts of Interest

The authors declare no conflicts of interest.

## Supporting information


**Table S1.** Prediction performance of machine learning models in the testing cohort.
**Figure S1.** LASSO algorithm of machine learning models in the testing cohort.

## Data Availability

Additional data supporting the results of this research can be available from the corresponding author, Zhao Shuang, upon reasonable request. Interested researchers are encouraged to contact the corresponding author to discuss data access conditions and potential collaborative opportunities.
