# Supplementary material for: Explainable Machine Learning Predictions for the Benefit From Chemotherapy in Advanced Non‐Small Cell Lung Cancer Without Available Targeted Mutations
Source: Clin Respir J. 2024 Dec 18;18(12):e70044. doi: 10.1111/crj.70044 (PMC11655385; doi:10.1111/crj.70044)

**SUPPLEMENTARY TABLE 1** Prediction performance of machine learning models in the testing cohort.

| **Model** | **AUC**  (95% CI) | **Sensitivity**  (95% CI) | **Specificity**  (95% CI) | **PPV**  (95% CI) | **NPV**  (95% CI) |
| --- | --- | --- | --- | --- | --- |
| Random forest (RF) | 0.757  (0.753~0.761) | 0.694  (0.69~0.0.698) | 0.738  (0.734~0.742) | 0.342  (0.338~0.346) | 0.927  (0.926~0.928) |
| eXtreme Gradient Boosting (Xgboost) | 0.78  (0.777~0.783) | 0.698  (0.694~0.702) | 0.735  (0.732~0.738) | 0.338  (0.334~0.342) | 0.928  (0.927~0.929) |
| Light Gradient Boosting Machine（LightGBM） | 0.733  (0.73~0.736) | 0.653  (0.649~0.657) | 0.704  (0.701~0.707) | 0.297  (0.294~0.3) | 0.914  (0.913~0.915) |
| Logistic regression (LR) | 0.677  (0.673~0.681) | 0.617  (0.613~0.621) | 0.652  (0.648~0.656) | 0.256  (0.253~0.259) | 0.899  (0.897~0.901) |

Abbreviations: AUC, area under the curves; PPV, positive predictive value; NPV, negative predictive value.

**SUPPLEMENTARY** **FIGURE 1** LASSO algorithm of machine learning models in the testing cohort.


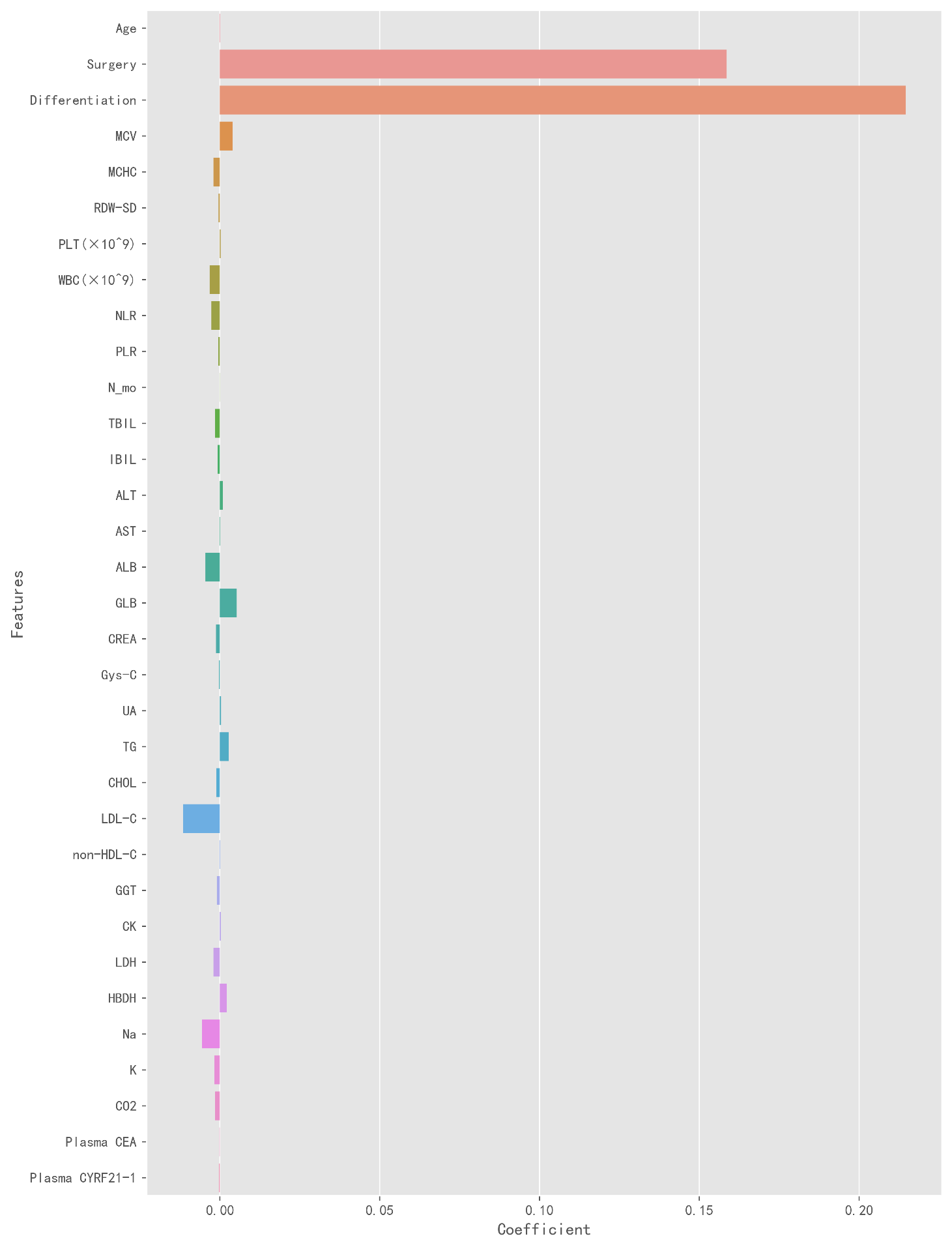

Supplement: Supplementary file 1 — Table S1. Prediction performance of machine learning models in the testing cohort. Figure S1. LASSO algorithm of machine learning models in the testing cohort. [file CRJ-18-e70044-s001.docx]
